# Efficient Surface Plasmon Polariton Excitation and Control over Outcoupling Mechanisms in Metal–Insulator–Metal Tunneling Junctions

**DOI:** 10.1002/advs.201900291

**Published:** 2020-02-22

**Authors:** Ksenia S. Makarenko, Thanh Xuan Hoang, Thorin J. Duffin, Andreea Radulescu, Vijith Kalathingal, Henri J. Lezec, Hong‐Son Chu, Christian A. Nijhuis

**Affiliations:** ^1^ Department of Chemistry National University of Singapore 3 Science Drive Singapore 117543 Singapore; ^2^ Department of Electronics and Photonics Institute of High Performance Computing A*STAR (Agency for Science, Technology and Research) 1 Fusionopolis Way, #16‐16 Connexis Singapore 138632 Singapore; ^3^ NUS Graduate School for Integrative Sciences and Engineering National University of Singapore 3 Science Drive Singapore 117543 Singapore; ^4^ NUSNNI‐NanoCore National University of Singapore Singapore 117411 Singapore; ^5^ Physical Measurement Laboratory National Institute of Standards and Technology Gaithersburg MD 20899 USA; ^6^ Centre for Advanced 2D Materials National University of Singapore 6 Science Drive 2 Singapore 117546 Singapore

**Keywords:** inelastic electron tunneling, light emission, roughness, surface plasmon polaritons, tunnel junctions

## Abstract

Surface plasmon polaritons (SPPs) are viable candidates for integration into on‐chip nano‐circuitry that allow access to high data bandwidths and low energy consumption. Metal–insulator–metal tunneling junctions (MIM‐TJs) have recently been shown to excite and detect SPPs electrically; however, experimentally measured efficiencies and outcoupling mechanisms are not fully understood. It is shown that the MIM‐TJ cavity SPP mode (MIM‐SPP) can outcouple via three pathways to i) photons via scattering of MIM‐SPP at the MIM–TJ interfaces, ii) SPPs at the metal–dielectric interfaces (bound‐SPPs) by mode coupling through the electrodes, and iii) photons and bound‐SPP modes by mode coupling at the MIM‐TJ edges. It is also shown that, for Al‐AlO*_x_*‐Cr‐Au MIM‐TJs on glass, the MIM‐SPP mode outcouples efficiently to bound‐SPPs through either electrode (pathway 2); this outcoupling pathway can be selectively turned on and off by changing the respective electrode thickness. Outcoupling at the MIM‐TJ edges (pathway 3) is efficient and sensitive to the edge topography, whereas most light emission originates from roughness‐induced scattering of the MIM‐SPP mode (pathway 1). Using an arbitrary roughness profile, it is demonstrated that various roughness facets can raise MIM‐SPP outcoupling efficiencies to 0.62%. These results pave the way for understanding the topographical parameters needed to develop CMOS‐compatible plasmonic circuitry elements.

## Graphical abstract

Selective electrical surface plasmon polariton excitation is demonstrated using metal–insulator–metal tunneling junctions by changing electrode thickness. Moreover, surface roughness is shown to enhance outcoupling by three orders of magnitude compared to previously believed possible, resolving a large controversy in the field, as well giving new insights into practical devices exploiting light–matter interactions in tunneling junctions for on‐chip applications.

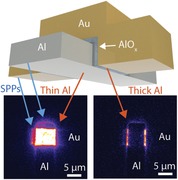

## 1 Introduction

**Figure 1:**
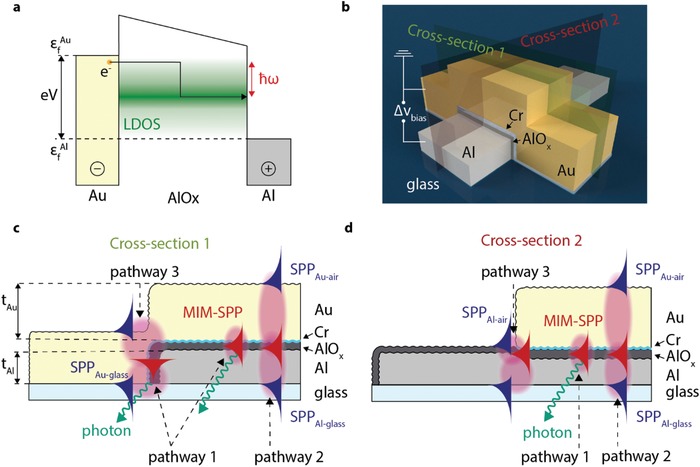
a) Energy level diagram of MIM‐TJ under applied bias. b) Schematic illustration of the MIM‐TJ (Al‐AlO*_x_*‐Cr‐Au) connected to Al and Au metal strips that serve as plasmonic waveguides. c,d) Schematic illustrations of the cross‐sections indicated in panel (b) showing the possible SPP modes at the metal–air/glass interfaces (in blue) and the MIM‐SPP mode (in red). The three MIM‐SPP outcoupling pathways are indicated with pathway 1: photon emission from scattering off of the MIM–TJ interface roughness, pathway 2: roughness‐enhanced momentum matched spatial mode overlap of the MIM‐SPP and various bound‐SPP modes, and pathway 3: MIM‐SPP outcoupling at the MIM‐TJ edge to photons and the bound‐SPP modes.

Sub‐diffraction limit optoelectronic circuitry has been demonstrated by integrating surface plasmon polaritons (SPPs)[qv: 1–3]—coherent, collective oscillations of free electrons propagating at metal–dielectric interfaces—into a myriad of applications in fields such as nano‐optics,[qv: 4–8] sensing,[qv: 9–11] sub‐wavelength imaging,[qv: 2,3] or nano‐optoelectronics.[qv: 12,13] SPPs are typically excited with bulky light sources that are too large for nano‐circuitry integration; hence, for many applications it would be desirable to have access to footprint‐compatible electrically driven SPP sources. Metal–insulator–metal tunneling junctions (MIM‐TJs) are known to electrically excite SPPs and photons via inelastic tunneling under an applied bias[qv: 14–17] (**Figure** **1**a) and are therefore interesting candidates.[qv: 18–20] Until recently, prohibitively low electron‐to‐photon conversion efficiency (10^4^–10^7^ electrons result in a single photon[qv: 14–16,21–23]) due to the large momentum mismatch between the initially excited MIM‐TJ cavity mode (MIM‐SPP) and the radiating mode, coupled with an inelastic tunneling event probability of 10%,[qv: 24] has prevented practical applications of MIM‐TJs. Recently, we have shown that the electron‐to‐SPP conversion efficiency can be experimentally as high as 1% in a planar Al‐AlO*_x_*‐Cr‐Au MIM‐TJ,[qv: 17] and Qian et al.[qv: 25] demonstrated 2% electron‐to‐photon emission efficiency in a resonant Ag–polymer–Ag nanocrystal tunnel junction, both within one order of magnitude agreement with theoretical inelastic tunneling models,[qv: 24,26] demonstrating the potential of MIM‐TJs in nano‐scale optoelectronics.

Despite this recent experimental evidence of efficient SPP excitation in MIM‐TJs, theoretical treatises of these systems continue to argue that these efficiencies cannot be due to outcoupling of the MIM‐SPP mode, and an alternative interpretation is needed.[qv: 27,28] In this work we demonstrate that, by modeling MIM–TJ interface surface roughness and electrode thickness with a simple ad hoc roughness model, electron‐to‐SPP efficiencies can be within an order of magnitude of experimental measurement. Furthermore, we demonstrate experimental control over outcoupling of the MIM‐SPP mode and the outcoupling pathways to radiative (photons) and non‐radiative (SPPs) modes. Our work shows experimental efficiencies can be further increased by exploiting surface roughness at the MIM–TJ interfaces[qv: 22,29,30] which, in principle, can be extended by coupling other types of structures to junctions, such as antennas or gratings.

The exact contributions from different SPP and photon excitation pathways from the MIM‐SPP mode remain experimentally unclear in MIM‐TJs.[qv: 26] MIM‐TJ excitation of radiating and non‐radiating modes can be split into two steps. Initially, the energy quanta donated from inelastically tunneling electron couples to the local density of optical states (LDOS—Figure 1a). The decay pathway of this energy is defined by the LDOS of the system, which preferentially couples to the cavity mode (MIM‐SPP) in Al‐AlO*_x_*‐Cr‐Au MIM‐TJs.[qv: 31–33] Once the MIM‐SPP is excited, it then outcouples to both the radiative and non‐radiative mode, as well as dissipating into the electrodes.[qv: 34] The MIM‐SPP mode has a wavevector of an order of magnitude larger than the single‐interface SPP modes that exist at the metal/dielectric interfaces (bound‐SPP modes) at visible/near‐IR frequencies. Parzefall et al.[qv: 27] recently theoretically investigated the outcoupling efficiency of the MIM‐SPP mode of an ideally smooth MIM‐TJ without surface roughness or topography, considering only outcoupling via pathway 2 (the only possible pathway in smooth systems; see the following sections), and reported very low outcoupling efficiencies on the order of 10^−8^. Therefore, in order to increase outcoupling efficiencies, momentum‐matching conditions at the metal–dielectric interfaces are required to outcouple the mode to both free space photons and to bound‐SPPs.[qv: 26,35] Randomly roughened electrodes,[qv: 17,22,29,30,36–42] gratings,[qv: 21,43,44] and prisms,[qv: 45] have been integrated into MIM‐TJs to preferentially outcouple the MIM‐SPP mode or the bound‐SPP mode(s) to photons by reducing this momentum mismatch.[qv: 17,26,43–46]

Root‐mean‐squared (RMS) surface roughness *σ_m_* enhances both mode overlap and momentum matching between the MIM‐SPP mode and the daughter modes. The mode overlap of the MIM‐SPP depends on the decay length δ_MIM_ – the distance that the MIM‐SPP extends into either electrode – which is typically tens of nanometers.[qv: 17,47] Mode overlap is only efficient when the thickness of the electrode *t_m_* is on the order of the δ_MIM_,[qv: 48,49] and even then requires a roughened topography to momentum match. Surface roughness enables momentum‐matching of the MIM‐SPP mode and the daughter mode by modifying the local permittivity *ε_m_′* (as a function of frequency ω) by *ε_m_′*(ω, *σ_m_*) = *ε_m_*(ω) + *δε_m_*(ω, *σ_m_*) where *δε_m_* is the local perturbation to *ε_m_* which modifies the local electromagnetic environment.[qv: 50–52] Dawson et al.[qv: 22] calculated that, for Al‐AlO*_x_*‐Au MIM‐TJs, the ideal *σ_m_* for the most efficient out‐coupling of the MIM‐SPP mode to photons is *σ_m_* ≈5 nm.[qv: 22,26] Moreover, recent studies into atomic‐scale topographical defects in metallic nanoparticle clusters that can act as plasmonic “picocavities” that can cause highly localized perturbations to the electromagnetic topography.[qv: 53,54] For the highly confined MIM‐SPP mode in a 2 nm MIM‐TJ, these atomic‐level defects could potentially have pronounced effects on localized Purcell enhancements as well as more efficient momentum‐matching to photons and bound‐SPPs, further increasing the efficiency of MIM‐SPP outcoupling.[qv: 53–56]

Here we show through experiments supported by theory that the MIM‐SPP mode readily outcouples via three possible outcoupling pathways of the MIM‐SPP mode: i) scattering of the MIM‐SPP to photons at the different dielectric–metal interfaces[qv: 16,38–41,44,49] (Figure 1c,d, pathway 1), ii) coupling of the MIM‐SPP to bound‐SPP modes by spatial mode‐overlap and roughness‐induced momentum matching[qv: 22,30,35,37,57] (Figure 1c,d, pathway 2), and iii) direct coupling of the MIM‐SPP mode to photons as well as the bound‐SPP modes at the edge of the MIM‐TJ and the adjacent waveguides[qv: 14,27] (Figure 1c,d, pathway 3). By studying light emission from Al‐AlO*_x_*‐Cr‐Au MIM‐TJs integrated into Al and Au waveguides (Figure 1b) and varying the Al and Au thickness (Figure 1c,d) we demonstrate that the MIM‐SPP mode couples to the bound‐SPP modes (Figure 1c,d), where the coupling efficiency is a function of the effective electrode thickness. Our simulations show that the majority of the bound‐SPP excitation originates from surface roughness‐induced momentum matching of the MIM‐SPP. The homogeneous light emission from the MIM‐TJ area originates from the scattering of the MIM‐SPP at the interfaces between the electrodes and the insulator layer. Our results uncover the origin of light emission from MIM‐TJs and give new insights into the contributions and control of the different MIM‐SPP mode outcoupling pathways, which serves to guide the rational design of future plasmonic‐electronic devices based on tunneling junctions.

## 2 Results and Discussion

### 2.1 Electrical Characterization

**Figure 2:**
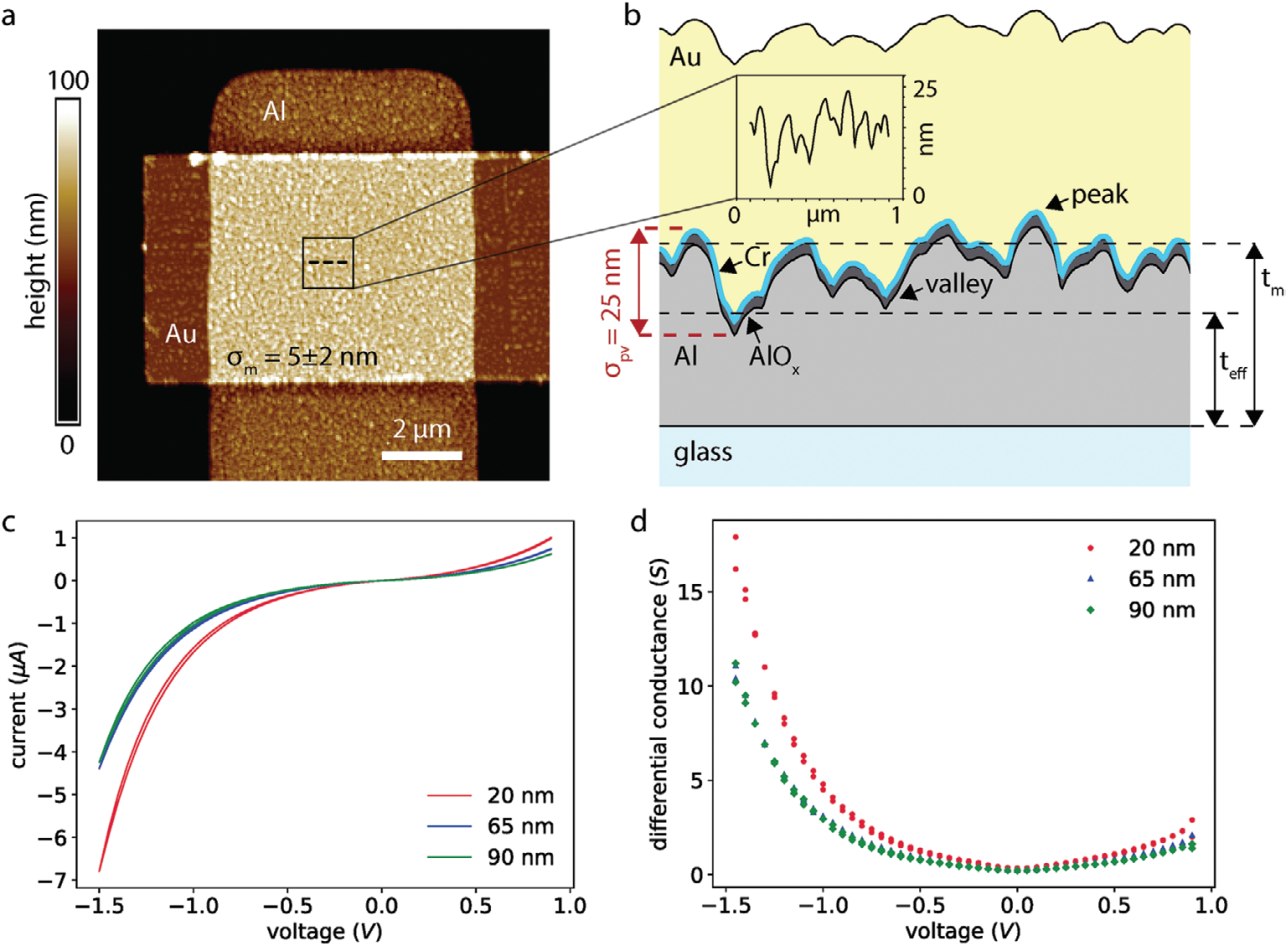
a) AFM image of a typical MIM‐TJ with *σ_m_* = 5±2 nm. b) Measured line scan of the MIM‐TJ as indicated by the dashed line indicated in (a) showing the *t*_eff_ and *t_m_*. c) *IV*‐characteristics of the MIM‐TJs of different thicknesses. d) Differential conductance d*I*/d*V* showing parabolic bias dependence.

The devices were fabricated on borosilicate coverslips following previously reported methods (see Section S1, Supporting Information).[qv: 17] **Figure** **2**a shows the AFM image from which we determined a value of *σ_m_* = 5 ± 2 nm for Al and *σ_m_* = 1.0 ± 0.2 nm for Au (both measured over an area of 1 × 1 µm^2^). Figure 2b shows a line scan recorded from the Al surface revealing peak‐to‐valley roughness σ_pv_ of 25 ± 2 nm. We fabricated four different MIM‐TJs with varying electrode thickness *t_m_*. A rough surface topography reduces the effective thickness of the electrodes by *t*_eff_ = *t_m_* – *σ_m_* as indicated in Figure 2b, which further increases the coupling efficiency of the MIM‐SPP mode[qv: 26,35,37] via pathway 2. **Table** **1** gives the measured electrode thickness and effective thickness for each electrode from the AFM line scans of the four samples. All deposition methods and rates were kept the same in order to keep surface roughness similar for all devices. The rms surface roughness of the glass coverslip (σ of 0.17 ± 0.01 nm measured over an area of 1 × 1 µm^2^, see Section S1, Supporting Information) is two orders of magnitude smaller than the roughness of the electrodes and thus negligible.

**Table 1:** Metal thicknesses and effective thicknesses for each sample

Sample	*t*_Al_ [nm]	*t*_Au_ [nm]	*t*_Al,eff_ [nm]	*t*_Au,eff_ [nm]
1	20	155	14 ± 2	149 ± 2
2	65	155	59 ± 2	149 ± 2
3	90	155	84 ± 2	149 ± 2
4	40	55	34 ± 2	49 ± 2

To ensure that the Al‐AlO*_x_*‐Cr‐Au MIM‐TJs were dominated by direct tunneling, the *IV*‐characteristics prior to the optical measurements (Figure 2c) were recorded. All *IV*‐curves show typical tunneling behavior with a positive, parabolic differential conductance (d*I*/d*V—* Figure 2d). Furthermore, we have previously shown that these MIM‐TJs have currents that scale with area and the *IV*‐curves are independent of temperature,[qv: 17] all of which satisfy the Rowell criteria and are indicative of junctions dominated by direct tunneling.[qv: 58,59] Moreover, all *IV*‐curves show similar currents, suggesting that the AlO*_x_* thickness is similar, which is crucial to directly compare photoemission intensities.

### 2.2 Optical Characterization—EMCCD Imaging

The optical properties of the MIM‐TJs were analyzed using an inverted microscope equipped with an EMCCD camera and UV–vis spectrometer using a 100× oil immersed objective with a numerical aperture NA = 1.49. The light emission from the MIM‐TJs was collected beneath the Al electrode and the glass substrate while applying a bias across the MIM‐TJ. The setup has been described in detail in ref. 17.

**Figure 3:**
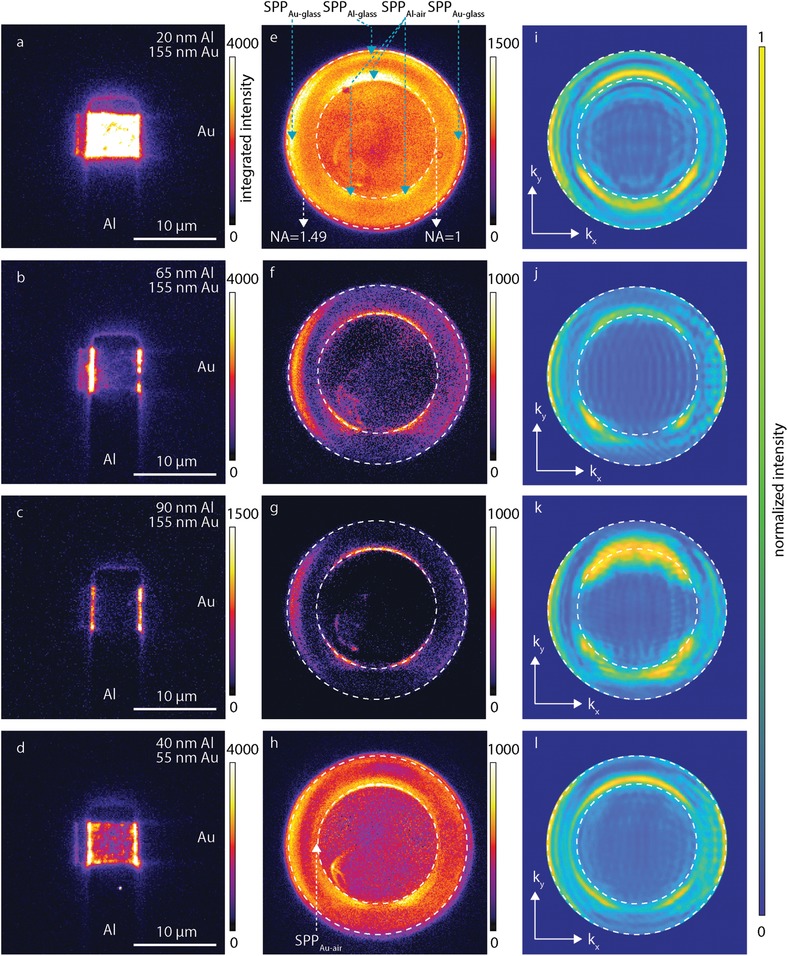
Wide‐field inverted optical microscopy images of the Al‐AlO*_x_*‐Cr‐Au MIM‐TJs with different electrodes thickness. EMCCD images of the devices measured at *V*_bias_ = −1.45 V with 155 nm thick Au top electrode and a) 20, b) 65, and c) 90 nm thick Al bottom electrode; d) device with 55 nm thick Au top and 40 nm thick bottom Al electrodes. e–h) Back focal plane (BFP) images of the corresponding MIM‐TJs; *V*_bias_ = −1.5 V for (e)–(f), −1.55 V for (g), and −1.6 V for (h), and corresponding normalized simulated BFP images in i–l). All EMCCD images have had their intensity individually scaled to best show MIM‐TJ light emission and scattering at the end of the waveguides. All BFP images have had their respective intensities scaled to give the best contrast. The inner white dashed line in the BFP images refers to the critical angle of the total internal reflection at the air–glass interface (*k*_SPP_ */k*_0_ = 1.0); the outer white dashed line indicates the maximum angle of the collected light limited by the NA of the objective (*k*_SPP_ */k*_0_ = 1.49).

**Figure** **3**a–c shows the real plane EMCCD images recorded from three different MIM‐TJs under applied bias as a function of the Al electrode thickness *t*_Al_ = 20, 65, and 90 nm; in this experiment we kept *t*_Au_ at 155 nm so that no modes at the Au–air interface could be excited (see the following sections). Each image intensity was scaled such to show any light emission from both the MIM‐TJ area and scattered bound‐SPPs at the end of the waveguides. From these three images, we make the following four observations: 1) light emission is visible from both the MIM‐TJ area and the ends of the Al and Au plasmonic waveguides, 2) light emission from the MIM‐TJ area decreases with increasing Al thickness, 3) light emission from the end of the waveguides decreases with increasing Al thickness, and 4) the light emission intensity is not homogeneous from the MIM‐TJ.

These observations can be explained as follows. The light emitted from the MIM‐TJ area is the result of scattering of the MIM‐SPP mode (pathway 1). The light emitted from the end of the waveguides is caused by scattering of the bound‐SPP modes, which were excited by the MIM‐SPP mode (pathway 2). *t*_eff_ for each MIM‐TJ is reduced by *σ_m_*, making all electrodes effectively thinner, enhancing mode overlap and increasing coupling efficiency. For the MIM‐TJ shown in Figure 3a, *t*_Al,eff_ = 15 nm and therefore the MIM‐SPP mode can readily outcouple via pathways 1 and 2 because *t*_Al,eff_ < δ_MIM_ (see Section 2.4) resulting in light emission from both the MIM‐TJ area and the ends of the waveguides. In contrast, as *t*_Al,eff_ becomes increasingly thicker than δ_MIM_, the mode overlap between the MIM‐SPP and SPP_Al‐glass_ decreases rapidly, and the MIM‐SPP mode cannot directly outcouple to photons, diminishing the outcoupling via pathways 1 and 2. Figure 3c shows that the MIM‐TJ area light emission when *t*_Al,eff_ = 85 nm is reduced by a factor of ≈20 compared to *t*_Al,eff_ = 15 nm, indicating that outcoupling via channel 1 is effectively quenched. This observation is in line with that reported by Kroó et al.[qv: 48] who reported complete quenching of the MIM‐SPP scattered emission at *t*_Ag_ > 80 nm in Al‐AlO_x_‐Ag MIM‐TJs. Light emission from the ends of both waveguides is also very weak, indicating that pathway 2 is also effectively quenched. Light emission from this MIM‐TJ is now dominated by scattering of the MIM‐SPP from the edges of the MIM‐TJ. Here the MIM‐SPP mode mainly outcouples to the bound‐SPPs by pathway 3.

Further indicating the role of surface roughness, the light intensity in Figure 3a–c varies spatially across the MIM‐TJ area and edges. This is because σ_pv_ ≈ δ_MIM_ in each MIM‐TJ, which implies that in areas where the Al is thickest (Figure 2b), the photons need to travel an extra factor of δ_MIM_ to reach the glass interface, leading to strong attenuation (see Figure S1, Supporting Information, for Al skin depth) relative to the photons close to the surface (valleys; Figure 2b). Hence, surface roughness can also explain the inhomogeneous light emission from the MIM‐TJ area.

Our results are not in agreement with the theoretical work of Parzefall et al.[qv: 14,27] who did not take the role of surface roughness into consideration and argued that light emission should mainly come from the MIM‐TJ edges (pathway 3) in smooth MIM‐TJs. They claim that due to the small MIM‐SPP propagation length *Λ*_MIM‐SPP_ ≈20 nm for optical frequencies, and small value of δ_MIM_ ≈20 nm, the MIM‐SPP mode can only outcouple to other modes at the MIM‐TJ edge (pathway 3), as MIM‐SPP modes excited further than *Λ*_MIM‐SPP_ would be attenuated. This leads them to conclude that MIM‐SPP out‐coupling efficiency should be very low (on the order of 10^−8^).[qv: 14,27] These works are accurate for smooth MIM‐TJs with no roughness but neglect to consider the role of surface roughness and coupling via pathway 2. Their argument cannot explain considerable literature that reports light emission from the MIM‐TJ area,[qv: 16,21,22,26,30,37,43–46,48,49,60–62] and cannot explain the observations shown in Figure 3.

### 2.3 Optical Characterization: BFP Imaging

To explore the outcoupling pathways described above in more detail, we recorded back focal plane (BFP) images to characterize the light emission from the entire device in *k*‐space as a ratio of SPP momentum *k*_SPP_ to the momentum of light in a vacuum *k*_0_, which splits up the collected intensity from light scattered directly from the MIM‐TJ (*k/k*_0_ < 1) and light from SPP scattering (*k/k*_0_ > 1). Figure 3e–g shows corresponding BFP images recorded from the MIM‐TJs as a function of *t*_Al,eff_. Figure 3e shows that light is not scattered discretely in all angles from the MIM‐TJ area, while the opposite is true for Figure 3g where only light emitted under discrete angles is visible. This observation supports the hypothesis that the MIM‐SPP mode outcoupling is dominated by pathways 1 and 2 for MIM‐TJs with *t*_Al,eff_ ≈ δ_MIM_ but not when *t*_Al,eff_ > δ_MIM_, where only outcoupling by pathway 3 is possible. Furthermore, the intensity background within *k/k*_0_ < 1 implies that only roughness‐scattered photons can be detected at these angles; no background would be expected for an MIM‐TJ with no roughness, and this background is only visible in Figure 3e,h (see Figure S4, Supporting Information).

The BFP images make it possible to identify all bound‐SPP modes as indicated in Figure 3e (supported by theory, see the following sections). The bound‐SPP modes propagating along the waveguides at the metal–glass interface can be observed via scattering at the end of the Au or Al waveguides (as in Figure 3e–h), but cannot be detected efficiently by leakage radiation due to limitations of the NA of our detector (NA = 1.49 < *k*_SPP_ */k*_0_).[qv: 17]

BFP imaging allows us to identify which bound‐SPP modes are excited as a function of the electrode thickness. As mentioned above, with increasing Al thickness the coupling efficiency of the MIM‐SPP mode to the SPP_Al‐glass_ mode is expected to decrease due to the *t*_Al,eff_ > δ_MIM_. Since we measure the optical properties of the MIM‐TJs from the back side of the MIM‐TJs, the optical contribution of the SPP_Al‐air_ mode excited by pathway 3 also diminishes with increasing Al thickness as the Al becomes increasingly opaque. The SPP_Al‐air_ mode is still visible in Figure 3g, but the MIM‐SPP mode is not since *t*_Al,eff_ >> δ_MIM_. The BFP images in Figure 3e–g show the presence of the SPP_Au‐glass_ mode, but the 155 nm thick electrode prevents coupling of the MIM‐SPP mode to the SPP_Au‐air_ mode (*t*_Au,eff_ >> δ_MIM_).

To see coupling of the MIM‐SPP mode to the SPP_Au‐air_ mode, an MIM‐TJ with *t*_Al,eff_ = 35 nm and *t*_Au,eff_ = 50 nm was fabricated (Figure 3d). The Al electrode thickness was chosen so that the MIM‐SPP mode would be still visible but not be too intense to saturate the detector, obscuring all bound‐SPP modes. As anticipated, we observed lower light intensity from the MIM‐TJ area due to scattering of the MIM‐SPP mode relative *t*_Al,eff_ = 15 nm Al electrode (Figure 3a) but higher than for the MIM‐TJ *t*_Al,eff_ = 60 nm (Figure 3b). However, now the SPP_Au‐air_ mode is clearly visible in the BFP image (Figure 3h), confirming that reducing the top electrode thickness allows excitation of the SPP_Au‐air_ mode by pathway 2.

### 2.4 Simulations

We performed numerical calculations (finite‐different time‐domain—FDTD—Lumerical software[qv: 63]) of the angular light distribution from an incident MIM‐SPP excitation as a function of *t*_Al,eff_ and *t*_Au,eff_ (Figure 3i–l; for full simulation details see Section S4, Supporting Information), which show good agreement with the experimentally collected BFP (Figure 3e–h). This agreement in BFP features for different bound‐SPP modes in both theory and experiment confirms that the MIM‐SPP mode is initially excited and then couples to all available bound‐SPP modes, and that this coupling efficiency depends on *t*_Al,eff_ and *t*_Au,eff_ as described in detail above and shown in Figure 1.

The above observations lead us to conclude that the bound‐SPP modes outcouple predominantly at the ends of the Au and Al waveguides, while the majority of the light emission in the MIM‐TJ area comes from the direct outcoupling to photons of the MIM‐SPP mode, scattering off of roughness in the MIM‐TJ. Furthermore, by ranging from *t*_Al,eff_ *, t*_Au,eff_ *≈ δ*_MIM_ to *t*_Al,eff_ *, t*_Au,eff_ *>> δ*_MIM_, we can control the outcoupling of the MIM‐SPP mode to all possible bound‐SPP modes.

### 2.5 MIM‐SPP Outcoupling Pathways

**Figure 4:**
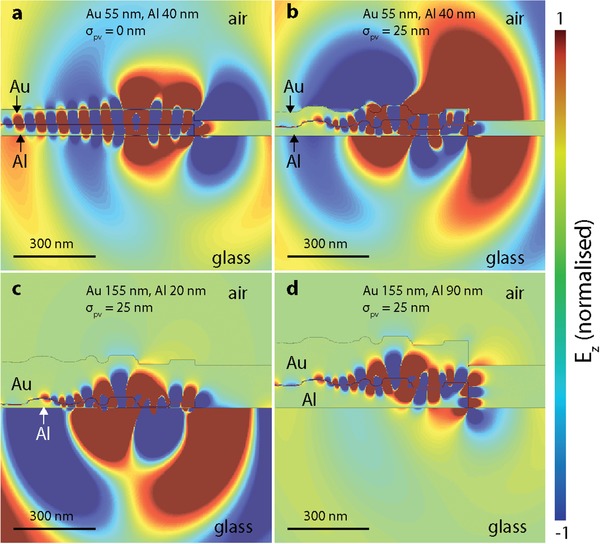
Mode profiles of MIM‐TJs for given experimental conditions: MIM‐TJ with Al thickness of 40 nm and Au thickness of 55 nm a) with σ_pv_ = 0 nm, and b) with σ_pv_ = 25 nm. c) MIM‐TJ with Al thickness of 20 nm and Au thickness of 155 nm and d) MIM‐TJ with Al thickness of 90 nm and Au thickness of 155 nm.

To demonstrate how the outcoupling of the MIM‐SPP mode can be affected by surface roughness and changes in *t*_eff_, we simulated the mode profile for MIM‐TJ structures with changes in the topography of the electrodes that represent various types of defects (see Section S4.4, Supporting Information for simulation details). Modeling roughness for highly confined plasmonic systems is notoriously complex due to nanoscale mode profiles.[qv: 50,64] Here, we only aim to show how a variety of local features in the electrode topography can affect the MIM‐SPP outcoupling pathways and improve the outcoupling efficiencies to the bound‐SPP modes. **Figure** **4** shows simulation results for MIM‐TJs with different roughness conditions and *t*_Al,eff_, where all thicknesses are indicated and the electric field intensity is normalized uniformly for all panels. Figure 4a shows the simulation results for a planar MIM‐TJ without roughness, and Figures 4b–d show the results for MIM‐TJs with surface defects with heights on the order of σ_pv_. Figure 4b shows the results for a σ_pv_ = 25 nm, Figure 4c shows an MIM‐TJ where σ_pv_ is close to *t*_Al,eff_, and Figure 4d shows an MIM‐TJ where σ_pv_ is ≈3.5× smaller than *t*_Al,eff_. Figure 4b shows the enhancement induced by the roughness of the outcoupling of the MIM‐SPP through the Al electrode by pathway 2 relative to the smooth electrodes in Figure 4a, where outcoupling is dominated at the MIM‐TJ edges via pathway 3. The presence of the roughened interface significantly increases outcoupling to all bound‐SPPs, supporting our observations in Figure 3h,i. Figure 4c,d shows the effect of thickness on pathway 2, where *t*_Al,eff_ *>> δ*_MIM_ significantly reduces the mode overlap of the MIM‐SPP and SPP_Al,glass_. This effect supports the observation in Figure 3c where only light from the MIM‐TJ edges can be observed. These mode profiles indicate the enhancement effect of both σ_pv_ and *t*_Al,eff_ on outcoupling via pathway 2, as well as demonstrate that pathway 3 remains relatively unaffected by either parameter.

### 2.6 MIM‐SPP Outcoupling Efficiencies

**Figure 5:**
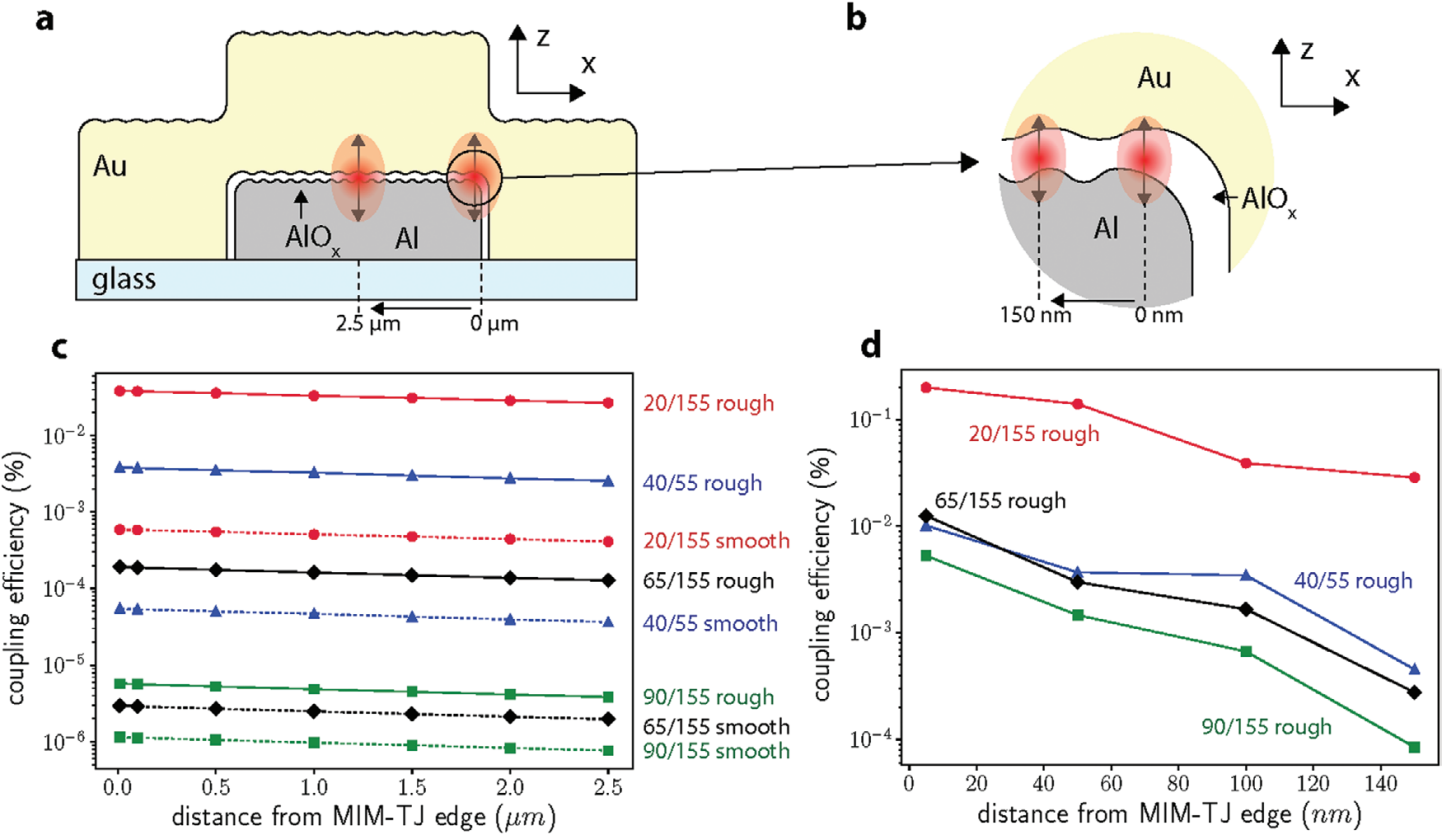
Schematics of indicating dipole positions for coupling simulations of the MIM‐SPP to the SPP_Au‐glass_ via a) pathway 2 and b) pathways 2 and 3. The simulated corresponding coupling efficiencies for the four different MIM‐TJs with a solid line indicating rough MIM‐TJs with *σ_m_* = 5 nm and a dashed line indicating smooth MIM‐TJs with *σ_m_* = 0 nm are shown for c) pathway 2 and d) pathways 2 and 3.

The outcoupling efficiencies of the MIM‐SPP mode to the bound‐SPP modes can be estimated by placing dipoles at different spatial positions inside MIM‐TJs of different thickness and roughness. **Figure** **5** shows the coupling efficiency of the MIM‐SPP to SPP_Au‐glass_ for an MIM‐TJ with *σ_m_* = 0 nm (dashed lines) and *σ_m_* = 5 nm (solid lines) for each corresponding MIM‐TJ thickness combination from Figure 3. Figure 5a,c shows the coupling efficiency between the two modes via pathway 2 for different dipole positions (indicated in Figure 5a) inside the insulator of the MIM‐TJ from the edge of the MIM‐TJ (*x* = 0 µm) to the center (*x* = 2.5 µm). Figure 5b,d shows the coupling efficiency for dipoles placed within 100 nm of the MIM‐TJ edge (indicated in Figure 5b), where edge effects begin to dominate due to contributions from pathways 2 and 3, as the distance from the edge approaches *x ≈Λ*_MIM‐SPP_ which we calculate to be *Λ*_MIM‐SPP_ ≈ 50 nm for λ = 900 nm (see Figure S4, Supporting Information). Since *Λ*_MIM‐SPP_ is a function of wavelength, this also implies that the contribution from pathway 3 will depend significantly on wavelength, and by extension a total outcoupling efficiency will also be wavelength dependent. We have chosen λ = 900 nm as this is the spectral peak of the emitted light from our MIM‐TJs (as reported previously[qv: 17]).

From Figure 5c,d we can see that for each MIM‐TJ, introduced surface roughness in the simulation increases the coupling efficiency of pathway 2 by ≈70×. This is due to the momentum mismatch in both pathways being reduced by scattering from the roughened surfaces. Furthermore, the thicker Al electrodes give weaker coupling efficiencies for both pathways, due to the reduction in spatial overlap of the modes. For pathway 2, as the dipole source approaches the edge of the waveguide (*x =* 0 µm) the distance the excited SPP_Al‐glass_ has to propagate is reduced, reducing propagation loss and increasing coupling efficiency. With these simulations we compute a total MIM‐SPP out‐coupling efficiency by all pathways of 0.62% for the thinnest Al electrode MIM‐TJ by integrating all contributions from pathways 2 and 3 across the MIM‐TJ, and then normalizing by MIM‐TJ area. Taking into consideration the proposed inelastic tunneling excitation efficiency of the MIM‐SPP mode of 10%,[qv: 24] we estimate a total electron‐to‐SPP efficiency of 0.062%. This efficiency is 4–6 orders of magnitude larger than recent publications predict,[qv: 14,27] and within an order of magnitude of our experimental findings.[qv: 17] We note that the improvements in the coupling efficiencies in the calculations only serve as estimates given our ad hoc roughness profile, currently we are conducting more comprehensive calculations with rigorously derived, statistically modeled surface topographies.

## 3 Conclusions

In this work, we have used imaging techniques coupled with FDTD simulation to understand SPP mode excitation mechanisms in MIM‐TJs, as well as developed basic structural understanding of MIM‐TJs to expose the influence of topographical surface roughness on MIM‐SPP outcoupling and discrete control over electrode thickness to “turn on and off” excitation of various bound‐SPPs as well as light emission. We demonstrate that in MIM‐TJs, inelastically tunneling electrons excite the MIM‐SPP mode, which then outcouples to electromagnetic modes by three pathways: i) outcoupling to photons by interface roughness‐induced momentum matching, ii) outcoupling to bound‐SPPs due to surface roughness both momentum matching and reducing the effective electrode thickness to increase spatial mode overlap, and iii) outcoupling directly to bound‐SPPs at the edge of the MIM‐TJ. We demonstrate that the efficiencies of pathways 1, 2, and 3 depend on the electrode thickness and that the contributions from each pathway can be controlled; when *t*_Al,eff_ *, t*_Au,eff_ *≈ δ*_MIM_ the coupling of the momentum‐matched, scattered MIM‐SPP mode is relatively efficient (pathways 1 and 2 contribute alongside pathway 3), and when *t*_Al,eff_ *, t*_Au,eff_ *> δ*_MIM_ the coupling is small (only pathway 3 contributes).

This MIM‐SPP out‐coupling can have efficiencies as high as 0.62%, confirming what we have claimed in previous works[qv: 17] that this process is similarly efficient as other on‐chip SPP excitation sources. We note that these estimates are conservative since we only modeled roughness coarsely as the main aim of this paper was to investigate the outcoupling mechanisms of the MIM‐SPP mode and the effect of different topographical defects. By changing the bottom and top electrode thickness in MIM‐TJs we can control the dominating mode and re‐direct outcoupling of the MIM‐SPP mode to the bound‐SPP mode either at the metal–glass or metal–air interfaces. Contrary to the recent arguments offered by Parzefall et al.,[qv: 14,27] we find that the contributions from the different MIM‐SPP outcoupling pathways vary depending on electrode thickness and interface roughness. Not only that, but we demonstrate that the majority of the light emanating from a statistically roughened MIM‐TJ area is indeed due to MIM‐SPP scattering from roughness at the electrode interfaces and outcoupling into photons, resolving a long‐standing question in MIM‐TJ literature. This research confirms the efficiency of MIM‐TJs as on‐chip SPP sources. Therefore we believe that MIM‐TJs, especially when combined with optical elements such as gratings,[qv: 65] antennas,[qv: 66] or waveguides,[qv: 17] are potentially interesting candidates for applications in sensing and opto‐electronics.

## Conflict of Interest

The authors declare no conflict of interest.

## Author Contributions

K.S.M., T.X.H., and T.J.D. contributed equally to this work. K.S.M. and A.R. fabricated the samples. K.S.M. performed the experiments and analysed the data. T.X.H. and H.‐S.C. performed theoretical calculations. V.K. provided theoretical support. T.J.D. assisted with measurements. C.A.N. conceived and designed the experiments. All authors discussed the results and commented on the manuscript.

## Supporting information

Supporting Information

Supplemental Video 1

Supplemental Video 2

Supplemental Video 3

Supplemental Video 4
