# Supplementary material for: Efficient Surface Plasmon Polariton Excitation and Control over Outcoupling Mechanisms in Metal–Insulator–Metal Tunneling Junctions
Source: Adv Sci (Weinh). 2020 Feb 22;7(8):1900291. doi: 10.1002/advs.201900291 (PMC7175257; doi:10.1002/advs.201900291)
Supplement: Supplementary file 1 — Supporting Information [file ADVS-7-1900291-s001.pdf]

Copyright WILEY-VCH Verlag GmbH & Co. KGaA, 69469 Weinheim, Germany, 2020.

ADVANCED SCIENCE
Open Access

Supporting Information

for *Adv. Sci.,* DOI: 10.1002/advs.201900291

Efficient Surface Plasmon Polariton Excitation and Control over Outcoupling Mechanisms in Metal–Insulator–Metal Tunneling Junctions

Ksenia S. Makarenko, Thanh Xuan Hoang, Thorin J. Duffin, Andreea Radulescu, Vijith Kalathingal, Henri J. Lezec, Hong-Son Chu, and Christian A. Nijhuis**

Supplementary Information

Efficient Surface Plasmon Polariton Excitation and Control over Outcoupling Mechanisms in Metal-Insulator-Metal Tunnelling Junctions

Ksenia S. Makarenko[†1], *Thanh Xuan Hoang*[†2], *Thorin J. Duffin*[†1, 3], *Andreea Radulescu*[1, 3], *Vijith Kalathingal*[1,4], *Henri J. Lezec*,[5] *Hong-Son Chu*[*2], *and Christian A. Nijhuis*[*1,3,4,6]

[1]Department of Chemistry, National University of Singapore, 3 Science Drive, Singapore 117543, Singapore

[2]Department of Electronics and Photonics, Institute of High Performance Computing, A*STAR (Agency for Science, Technology and Research), 1 Fusionopolis Way, #16-16 Connexis, Singapore 138632, Singapore

[3]NUS Graduate School for Integrative Sciences and Engineering, National University of Singapore, 3 Science Drive, Singapore 117543, Singapore

[4]NUSNNI-NanoCore, National University of Singapore, Singapore 117411, Singapore

[5]Center for Nanoscale Science and Technology, National Institute of Standards and Technology, Gaithersburg, Maryland 20899, United States

[6]Centre for Advanced 2D Materials, National University of Singapore, 6 Science Drive 2, Singapore 117546, Singapore

[†]These authors contributed equally to this work

*Authors to whom correspondence should be addressed: chmnca@nus.edu.sg, chuhs@ihpc.a-star.edu.sg

1. Device Fabrication.

The Al-AlO_x-Cr-Au MIM-TJs were designed and fabricated in a similar method to our previous work.[1] Al was used because it natively oxidises forming a tunnelling barrier and Au was chosen due to wide availability, low plasmonic losses and it is well-established in laboratory fabrication techniques. All devices were fabricated on borosilicate coverslips (Paul Marienfeld GmbH, 22×22 mm, 0.16-0.19 mm thick) with a roughness σ of 0.17 ± 0.01 nm measured over an area of 1 × 1 μm^2 – see Fig. S1 – which is two orders of magnitude smaller than the roughness introduced by thermal metal deposition.

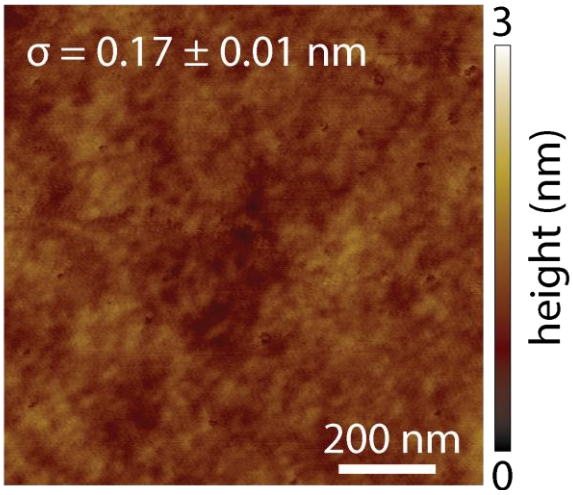

Figure S1. AFM image of the borosilicate substrates with the roughness indicated, measured over over an area of 1 × 1 μm^2.

A three-step fabrication process was performed. First, contact pads (100×80 μm) were patterned using a laserwriter (Microtech, LW405B), with bi-layer resist with a total resist thickness of ~1.3 μm: LOR 3A, (prebake at 170˚C for 5 min) and S1805 (prebake at 115˚C for 1 min) exposed with a 405nm beam at 320 mJ/cm^2. This was followed by the thermal deposition (Kurt J. Lesker, NANO 36) of the bi-metal layer of Cr/Au (3/25 nm) where Cr serves as an adhesion layer. In the second step after lift-off, the electrodes were patterned again by laserwriter and the Al layer of different thicknesses (t_{Al}=40, 80, 100 and 60 nm)

were thermally deposited. Finally, the top electrodes were exposed using electron beam lithography (JEOL, JBX-6300FS), using PMMA 950 A4 resist (MicroChem, prebake at 180˚C for 2 min) exposed with 5 nA current. After resist development, samples were immersed into MF-319 optical developer, with active ingredient TMAH which is an Al etchant, to remove AlOx for 20s and rinsed with water for 1 min. Etching thins the Al electrodes in the region under the top electrode, so that the final thickness in the MIM-TJ area (5×5 μm^2) is t_{Al}=20, 65, 90 and 40 nm. Fresh native AlO_x was then grown in ambient conditions for 1.5 h, before the Au layer with 1 nm of Cr adhesion layer was deposited using thermal evaporator (t_{Au}=155, 155, 155 and 40 nm). The first three samples all had top electrode fabricated at once to minimise substrate-to-substrate variation.

2. Electrical Measurements.

Prior to the light emission measurements, *IV*-characteristics were taken in order to confirm tunnelling behaviour of the MIMTJs. MIM-TJ voltage measurements were conducted using micromanipulators (Signatone) with Tungsten probes (ZN50R DC/RF) were performed using a source meter (Keithley 6430, Keithley Instruments) and controlled by homemade LabView programme. During all the experiments, the Au electrodes were grounded and the Al electrodes were biased. We have already shown previously[1] that Al-AlO_x-Cr-Au junction have lower break down at positive bias than at negative bias. Therefore, we applied voltage between +1 and -1.5 V with a step of 50 mV. Obtained *IV*-curves do not depend on the thickness of the bottom electrode (Fig. 2c). The differential conductance (d*I*/d*V*) plot in Fig. 2d demonstrates the parabolic bias dependence of tunnelling, one of the

Rowell criteria to identify tunnelling.[2] Our electrical measurements are representative of those that we have previously reported.[1]

3. Optical Measurements.

3.1 Optical setup. An inverted optical microscope (Nikon Eclipse Ti-*E*) equipped with an Andor spectrometer (Shamrock 122 303i) and an electron multiplying CCD (EMCCD, iXon Ultra 897) were used for the optical characterisation of the emitted light from the tunnelling junctions. Optical measurements were measured from the glass (back) side using a 100× oil objective (Numerical Aperture, NA=1.49). EMCCD images (both real and back focal planes) were taken with a 2 min integration time and 300 EM gain.

3.2 Al transmission measurements. The optical contribution to the EMCCD images (both real and BFP) from the $SPP_{Al\text{-}air}$ mode decrease with thickness of the Al. We performed transmission measurements on the metal to quantify this reduction and found that the Al has a experimentally derived skin depth δ_m = 23.2 nm at λ = 900 nm, and an optical penetration length of δ_γ = 11.6 nm at λ = 900 nm (Fig. S1) by fitting the data to 1/e power law. These transmission measurements are consistent with literature[3,4].

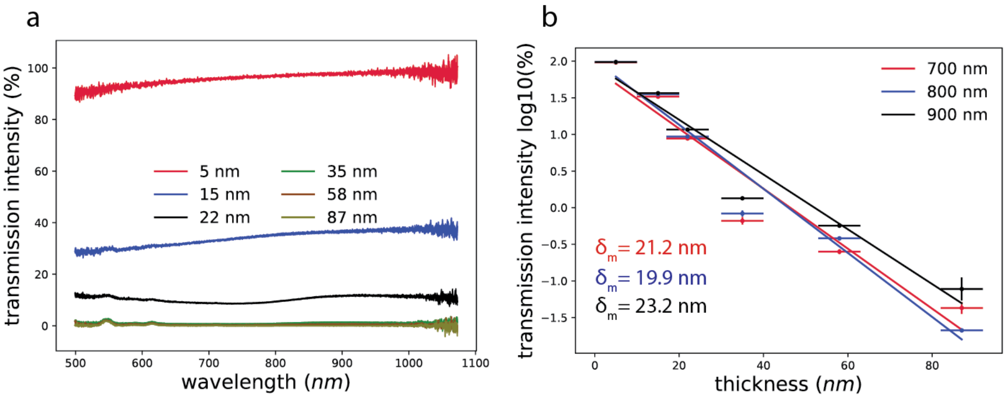

Figure S2. Al transmission measurements as a function of (**a**) wavelength and (**b**) as a function of wavelength with linear fit plotted and the optical decay length indicated.

4. Numerical calculations.

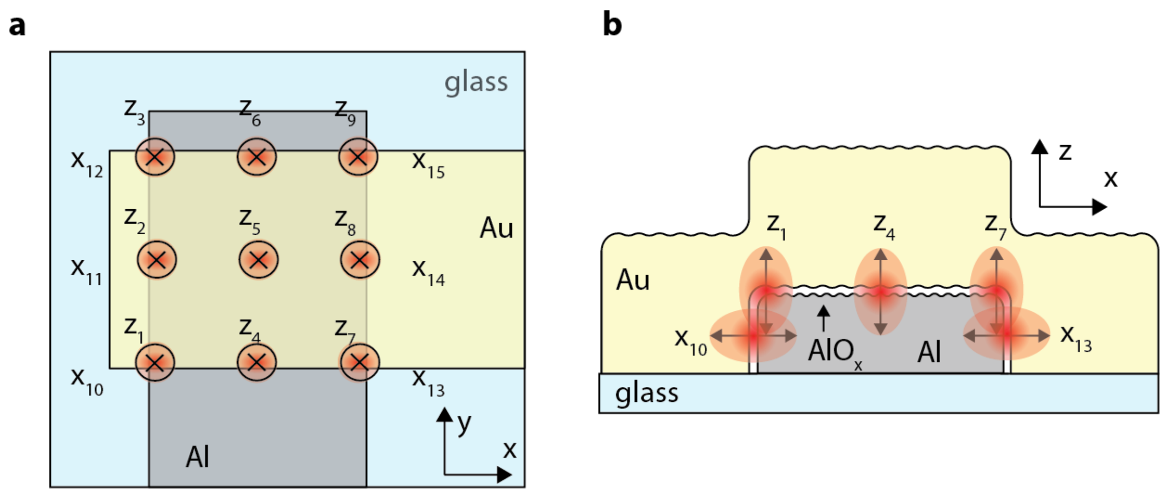

Figure S3. Schematic illustration of the m = 15 dipole source locations that mimic the MIM-SPP excitation mechanism in the MIM-TJs. (**a**) Top and (**b**) side view of MIM-TJ.

4.1 Dipole placement. For the BFP calculations in Fig. 3i-j, we performed numerical calculations (finite-difference time-domain – FDTD – Lumerical software[5]) of the angular light distribution as a function of $t_{Al,eff}$ and $t_{Au,eff}$ detailed as follows. MIM-TJ tunnelling currents are typically modelled using the dipolar approximation.[6,7] We used 15 dipole source locations (Fig. S2) to excite all the possible modes supported by the MIM-TJ. To justify the dipole location choices, we first consider the physical properties of the system. Surface roughness is due to the variation in Au surface grain distribution. These grains, as we can see in the AFM image in Fig. 2, generally have a width and height of a few tens of nanometres, which is on the same order as the MIM-SPP propagation length. This implies that for any MIM-SPP mode that is excited there will be an interaction with the neighbouring grain which creates an aggregate effect where although the MIM-TJ has varying surface features, the induced MIM-SPP modes will have similar properties and can be treated as the same across the surface.

In each dipole location, the source is modelled as an oscillating dipole that is aligned along the surface normal which coincides with the direction of the applied field. Each of these dipoles radiates electromagnetic energy and locally excites the MIM-SPP mode. For each wavelength of the dipole source, using the near-to-far-field-projection function of the software, we can evaluate the far-field intensity of the propagating light as it approaches the lens $I(\lambda, f, \theta, \varphi)$ – where f is the focal length of the objective, θ is the transverse angle and φ is the azimuthal angle at the focal point of the microscope objective. Considering the microscope objective lens (NA = 1.49) and the refractive index of the glass substrate (n = 1.52), the corresponding collected intensity by the lens is $I(\lambda) = I(\lambda, f, \theta, \varphi)\ /cos(\theta)$ where only the light approaching the lens with the incident angle $\theta < \theta_{\mathrm{m}}$ that satisfies $sin(\theta_m)=NA/n$ is collected. The $\cos(\theta)$ represents the apodisation effect of the collection lens. The Fourier image of the MIM-TJ is evaluated for each source position by integrating the intensity across all wavelengths $I(\lambda)$. The final Fourier images are obtained as a summation of the individual Fourier images as follows:

$$I_s = \sum_{m=1}^{15} \alpha_m \int_\lambda I_m(\lambda) d\lambda, \qquad \text{(S1)}$$

where a_m represents the total MIM-TJ area. All simulations were done over a 3D domain with volume of 16 × 16 × 2 μm^3. These dimensions incorporate the entire 5 × 5 μm^2 MIM-TJ with 3 μm truncated waveguides extending from each side.

4.2 Angular light emission. To understand the noise background in the BFP images (Fig. 3i-j) we simulated the angular distribution of light emission from the MIM-TJ (Fig. S3). Fig. S3 indicates that, for an MIM-TJ with a smooth interface (σ_{pv} = 0 nm), no signal in the BFP would be expected for the partial solid angle corresponding to $240^{\circ} < \phi < 300^{\circ}$ and $0^{\circ} < \theta < 30^{\circ}$ for an MIM-TJ of any thickness combination. Therefore, the signal in Fig. 3e within

$k_{xy}/k_0 < 1$ is direct evidence of photon scattering in the MIM-TJ from the surface roughness. The light emission in Fig. S3 corresponds to a dipole at *z5* from Fig. S2.

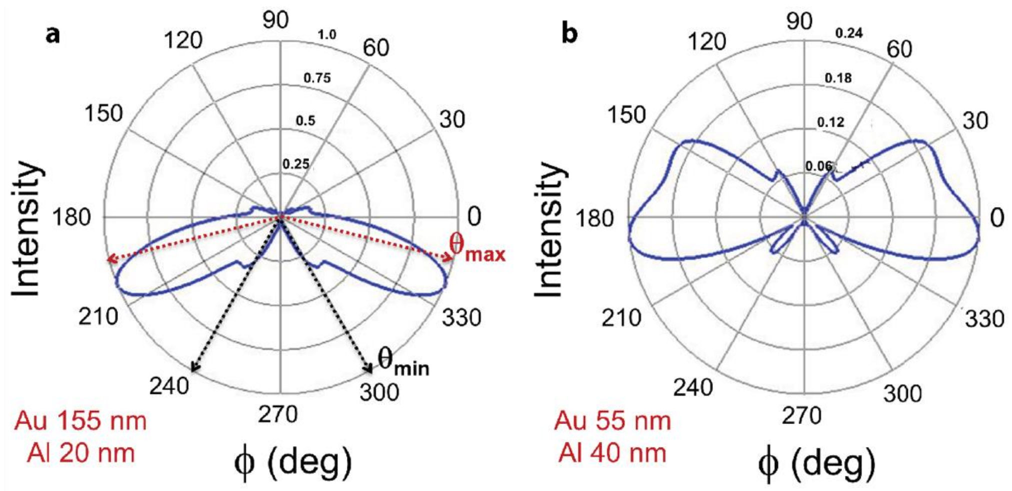

Figure S4. Angular light emission simulations from a dipole at location *z5* for electrodes of different thicknesses. Note the light emission always scatters outside an angle of 30°.

4.3 MIM-SPP propagation length. By simulating the dispersion of the MIM-SPP, we determine $\Lambda_{MIM\text{-}SPP}$. Fig. S4 shows the propagation constants $k_{MIM\text{-}SPP}$ and $\Lambda_{MIM\text{-}SPP}$ of the Au/AlOx/Al MIM-SPP mode from λ = 600 to 1000 nm. The propagation constants of the MIM-SPP mode (Fig. S4b) are much larger than the detectable values (limited by the numerical aperture (NA=1.49) of our oil objective), which means that we cannot directly detect this MIM-SPP mode. On the other hand, the propagation distance of this MIM-SPP mode is much smaller (18-24×) than the corresponding wavelength as shown in Fig. S4a.

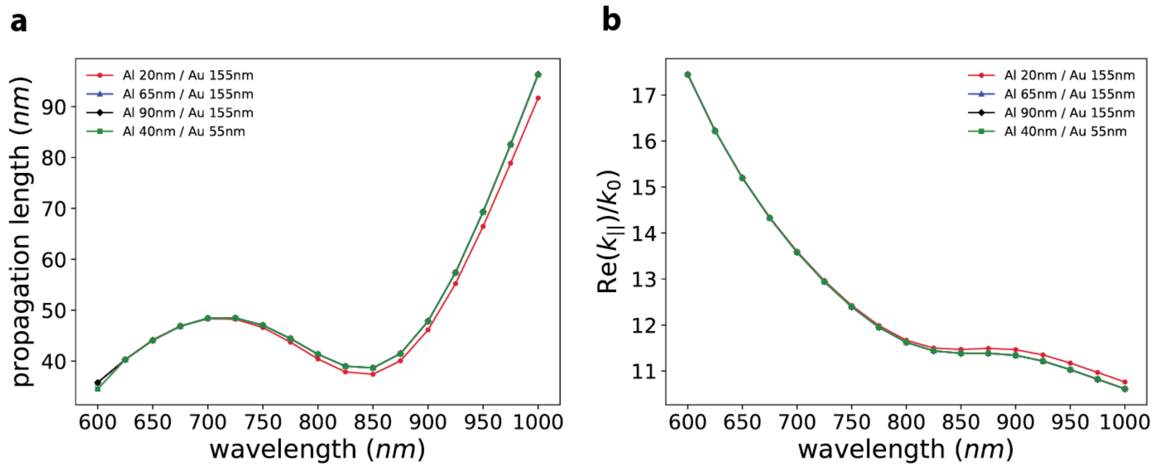

Figure S5. Simulation results of (**a**) $\Lambda_{MIM\text{-}SPP}$ and (**b**) corresponding $k_{MIM\text{-}SPP}$.

4.4 Mode profiles. The mode profiles shown in Fig. 4 demonstrate the effects on outcoupling by σ_{pv} (Fig. 4a,b) and t_{eff} (Fig. 4c,d). Attached are 4 videos demonstrating the evolution of the MIM-SPP to bound-SPP outcoupling over time. These videos show the modes from Fig. 4 in the main text as they couple from the initial dipole excitation. Video 1 shows the mode for the case when t_{Al} = 40 nm, t_{Au} = 55 nm and σ_{pv} = 0 nm. Video 2 shows the same MIM-TJ thickness but with σ_{pv} = 25 nm. Video 3 shows t_{Al} = 20 nm, t_{Au} = 155 nm and σ_{pv} = 25 nm, with t_{Al} increased to 90 nm in video 4. Due to the propagation distance of the MIM-SPP is in the range of 40-50 nm for λ = 900 nm, the outcoupling of the MIM-SPPs generated in the central area of the MIM-TJs is through the pathway 2 only. Fig. S5 shows the mode profiles similar to Fig. 4 except that we have excluded the edge so that only the outcoupling mechanism through the pathway 2 is present to emphasise the role of roughness. For the smooth MIM in Fig. S5a and the thick-electrode MIM in Fig. S5d the MIM-SPPs are localised to the MIM-TJs and are not efficiently coupled out to the other modes. However, with the roughness introduced into the tunnelling junctions and thinner electrodes in Fig. S5b,c the outcoupling enhancement is clearly appreciated.

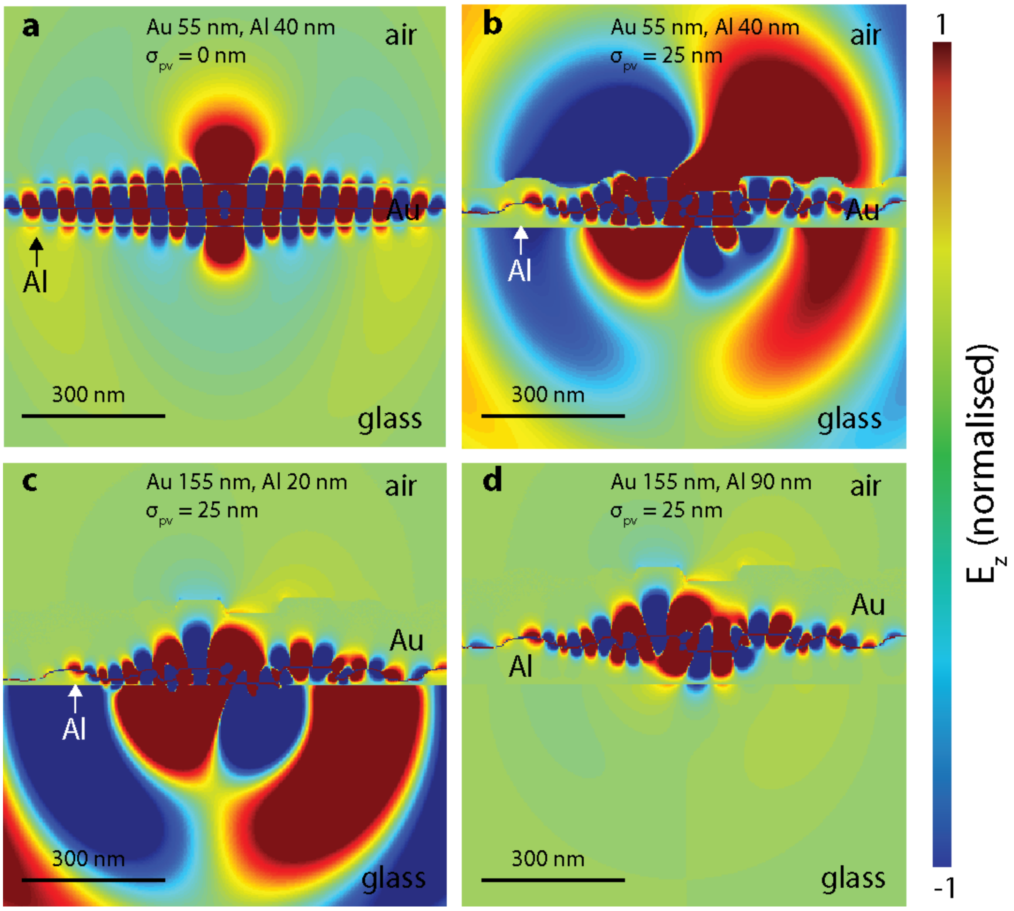

Figure S6. SPP mode profiles for different σ_{pv} and t_{eff}. **(a)** Smooth MIM-TJ with the Au and Al thicknesses of 55 nm and 40 nm, as in Fig. 3d. Without the roughness, the outcoupling is weaker than the same thickness in **(b)** but with σ_{pv} = 25 nm introduced into the simulation, the outcoupling is greatly enhanced. **(c)** Changing the electrode thickness to match Fig. 3a with the same roughness profile in **(b)** but $t_{eff,Al}$ is reduced so that we increase the spatial overlap of the MIM-SPP and the bound SPPs. The large spatial overlap results in the higher efficiency of the out-coupling through pathway 2. **(d)** For the opposite extreme, as in Fig. 3c we increase the Al thickness to 90 nm so that we can observe the attenuation effect of a thick electrode. Due to the small spatial overlap, even with the presence of the roughness the MIM-SPP can not be coupled out efficiently.

4.5 Efficiency Evaluation. We quantitatively study the coupling efficiencies of the MIM-SPP mode to the bound-SPP modes, taking into account the electrode thickness and

roughness. For each of the dipole positions, we collect the total power dissipated by the dipole P and the corresponding power coupled out into the forms of photon $P\gamma$ and bound-SPP P_{spp}. The out-coupling efficiency from the MIM-SPP to the bound SPP is then calculated by the ratio of P_{spp}/P. In the FDTD Solutions of Lumerical, we estimate the total power P by using a transmission box with the centre coinciding with the position of the dipole. The dimensions of the box are 2×2×2 nm^3 so that the box is placed entirely inside the insulator layer and hence there is no absorption inside the box. The net power flowing out of the box is hence equal to the total power dissipated by the dipole. The mesh size for the insulator layer is 0.25×0.25×0.25 nm^3. For the photon and bound SPP powers, we estimate them as follows. First, a 2D monitor with the dimensions of 16×16 μm^2 is placed in the substrate at a distance of 0.8 µm below the Al-glass interface for collecting photons radiated from the MIM-TJs. For the device with the gold electrode, another similar monitor is placed in the air region for the estimation of the photon power radiated into the upper half space. These monitors collect power radiated into a semi-angle collection angle of $\tan^{-1}(8/0.8) \approx 84^{\circ}$. Since the propagation distance of the MIM-SPP is in the range of 38 to 48 nm as shown in Fig. S3b, no MIM-SPP can reach a distance of 1 µm away from its generated position. Therefore, only the out-coupling bound SPPs and the photons can flow out of a transmission box with the centre coinciding with the dipole position and the dimensions of 2×2×1 μm^3. The net power flowing out of this transmission box $P_{\gamma\text{-}spp} = P_{\gamma} + P_{spp}$ is therefore the total power radiated into the forms of the photons and bound-SPPs. We can then estimate the power radiated into the form of the bound SPPs as $P_{spp} = P_{\gamma\text{-}spp} - P_{\gamma}$. The coupling efficiency is subsequently calculated by integrating all contributions over MIM-TJ, and then normalising by MIM-TJ area, and are presented in Fig. 5 in the main text. The roughness profile is assumed to comprise Al and Au grains. These grains are modelled as half ellipsoids randomly generated around the dipole with the radii in the range of 25 to 50 nm and the heights in the range of 7.5 to 12.5 nm.

These parameters are chosen so that a peak-to-valley roughness σ_{pv} = 25 nm and the statistical root-mean-square roughness scale σ_{vm} = 5 nm are modelled in accordance with our reported experimental measurements.

References

1. Du, W., Wang, T., Chu, H.-S. & Nijhuis, C. A. Highly efficient on-chip direct electronic–plasmonic transducers. *Nat. Photonics* **11**, 623–627 (2017).
2. Brinkman, W. F., Dynes, R. C. & Rowell, J. M. Tunneling Conductance of Asymmetrical Barriers. *J. Appl. Phys.* **41**, 1915–1921 (1970).
3. Dawson, P., Walmsley, D. G., Quinn, H. A. & Ferguson, A. J. L. Observation and explanation of light-emission spectra from statistically rough Cu, Ag, and Au tunnel junctions. *Phys. Rev. B* **30**, 3164–3178 (1984).
4. Semaltianos, N. G. Thermally evaporated aluminium thin films. *Appl. Surf. Sci.* **183**, 223–229 (2001).
5. Lumerical. Lumerical. (2018).
6. Parzefall, M. & Novotny, L. Light at the end of the tunnel. *ACS Photonics* **5**, 4195–4202 (2018).
7. Esteban, R., Borisov, A. G., Nordlander, P. & Aizpurua, J. Bridging quantum and classical plasmonics with a quantum-corrected model. *Nat. Commun.* **3**, 825 (2012).
